# Large artery atherosclerotic versus cardioembolism subtypes of large hemispheric infarction in the middle cerebral artery

**DOI:** 10.1007/s10072-025-08347-9

**Published:** 2025-07-12

**Authors:** Han Xiao, Zi-Yan He, Xue-Ming Li, Jing Mao, Yun Zhou

**Affiliations:** 1 Department of Rehabilitation Medicine, The Second Affiliated Hospital of Anhui Medical University, Hefei, 230601 Anhui China; 2 Research Center for Translational Medicine, The Second Affiliated Hospital of Anhui Medical University, Hefei, 230601 Anhui China; 3 Department of Rehabilitation Medicine, 901th Hospital of PLA, Hefei, 230031 Anhui China

**Keywords:** Large hemispheric infarction, Large artery atherosclerosis, Cardioembolism, TOAST, Retrospective study

## Abstract

**Background:**

Large hemispheric infarction (LHI) of the middle cerebral artery (MCA) is linked to high mortality and morbidity. This study aims to investigate the characteristics of large artery atherosclerosis (LAA) and cardioembolism subtypes of LHI in MCA.

**Methods:**

This retrospective cohort study included 70 patients with LHI hospitalized at the Second Affiliated Hospital of Anhui Medical University from May 2019 to May 2021. Patients were classified according to the TOAST classification into LAA and cardioembolism subtypes.

**Results:**

Among the 70 patients, 44 were identified with the LAA subtype (aged 76.00 years, 50% were male) and 26 with cardioembolism (aged 71.50 years, 57.1% were male). The LAA group exhibited significantly higher rates of hyperhomocysteinemia (18.2% vs. 0%, *P* = 0.022) and diabetes (38.6% vs. 15.4%, *P* = 0.042). In contrast, atrial fibrillation prevalence was higher in the cardioembolism group (84.6% vs. 20.5%, *P* < 0.001), as was the rate of decompressive craniectomy (15.4% vs. 2.3%, *P* = 0.041), while, hypertension prevalence, thrombectomy, and rehabilitation scores, showed no significant differences (all *P* > 0.05). Additionally, multivariable linear regression analysis showed that, after adjusted the confounders, LAA (vs. CE) subtype was independently associated with higher mRS scores (β = 0.86, 95%CI: 0.61–1.22), higher NIHSS (β = 4.85, 95%CI: 0.19–9.89), higher Visual Analog Scale (VAS) (β = 0.86, 95%CI: 0.66–1.12), and higher GCS (β = 0.62, 95%CI: 0.09-4.00) (all *P* < 0.05).

**Conclusions:**

Patients with the LAA subtype of LHI in MCA are more likely to have hyperhomocysteinemia and diabetes, while atrial fibrillation and the need for decompressive craniectomy are more prevalent in the cardioembolism subtype. LHI subtypes may significantly impact patient rehabilitation outcomes.

**Supplementary Information:**

The online version contains supplementary material available at 10.1007/s10072-025-08347-9.

## Background

Large hemispheric infarction (LHI) is a severe form of ischemic stroke manifesting as severe hemispheric syndrome (hemiparesis, gaze deviation, and cortical signs) followed by headache, vomiting, papilledema, and altered consciousness due to mass effect [1]. The annual incidence of LHI is 10–20 per 100,000, and mortality is 80% when conservative management is used.^2, 3^ LHI involves space-occupying cerebral edema that leads to rapid neurological decompensation resulting from cerebral infarction involving a large portion of a hemisphere, usually due to infarction of the entire middle cerebral artery (MCA) territory [2, 3]. 

Effective conservative treatment for LHI remains unresolved and is currently the same as for stroke (i.e., hypothermia and osmotic therapy). Early (< 24 h after onset) decompressive hemicraniectomy (DHC) can reduce mortality, but this reduction in mortality is accompanied by an increase in moderate to severe disability among many survivors [2, 4], placing a significant burden on families and society. Authoritative organizations recommend considering DHC in selected patients with symptomatic brain swelling or high risk, such as with extensive MCA territory ischemic stroke [5, 6]. According to the Trial of ORG 10,172 in Acute Stroke Treatment (TOAST) classification, the etiology of LHI can be divided into large artery atherosclerosis (LAA), cardioembolic (CE), small artery occlusion (SAO), other determined causes, and undetermined causes [7], with LAA and CE LHIs accounting for the majority of cases. Different outcomes among TOAST subtypes are due to differences in the cause and severity of the stroke [8], and there is currently little research on the characteristics of these two subtypes of LHI.

Therefore, this study aimed to explore the similarities and differences between the LAA and CE subtypes of LHI in MCA through a retrospective analysis of their clinical characteristics, imaging, comorbidities, complications, clinical presentation, treatment, and outcomes. The results could help provide references for clinical decision-making for patients with LHI.

## Methods

### Study design and patients

This retrospective cohort study included consecutive patients hospitalized with LHI between May 2019 and May 2021 at the Second Affiliated Hospital of Anhui Medical University. The hospital has a designated stroke center with a standardized ischemic stroke management protocol (Figure S1). Comparative analysis revealed no significant differences in clinical characteristics or outcomes between ER and non-ER admissions.

The inclusion criteria were: (1) diagnosis of MCA infarction based on clinical features infarction and imaging [9], (2) met the diagnosis criteria of LHI [10–12], and (3) classification as LAA or cardioembolic (CE) subtype according to the TOAST criteria [7]. Patients with posterior circulation infarcts or missing imaging data were excluded.

The diagnosis of LHI required the infarction met at least one of the following criteria: (1) infarction involving at least two-thirds of the MCA territory; (2) infarction volume ≥ 70 cm^3^ of irreversibly damaged neuronal tissue confirmed by flumazenil positron emission tomography; (3) infarction volume > 82 mL on diffusion-weighted magnetic resonance imaging (DWI) [10–12]. 

This study was approved by the Medical Research Ethics Management Committee of the Second Affiliated Hospital of Anhui Medical University. The requirement for individual informed consent was waived due to the retrospective nature of the study.

### Data collection and definitions

General information, including sex, age, insurance category, smoking and drinking status, infarction site, and handedness, was collected from medical records. Information on four common comorbidities in patients with LHI (i.e., hypertension, diabetes, hyperhomocysteinemia, and atrial fibrillation), five common complications (pulmonary infection, electrolyte disorders, urinary incontinence, urinary tract infection, and constipation), clinical presentation (muscle strength, sensation, pathological signs, hemorrhagic transformation after cerebral infarction, and malignant cerebral edema), treatment (thrombolysis, thrombectomy, DHC, and antiplatelet therapy), rehabilitation consultation, rehabilitation treatment, and outcomes were also collected.

#### Cranial imaging and ASPECTS scoring

Routine cranial CT scan parameters during the study period included a slice thickness of 5 mm, a pitch of 0.55, and an interslice gap of 192 × 0.6 mm. The routine cranial magnetic resonance imaging (MRI) scan parameters were a repetition time of 4300 ms, echo time of 101 ms, matrix of 240 × 240, slice thickness of 5 mm, interslice gap of 1 mm, and 24 axial slices.

**Fig. 1:**
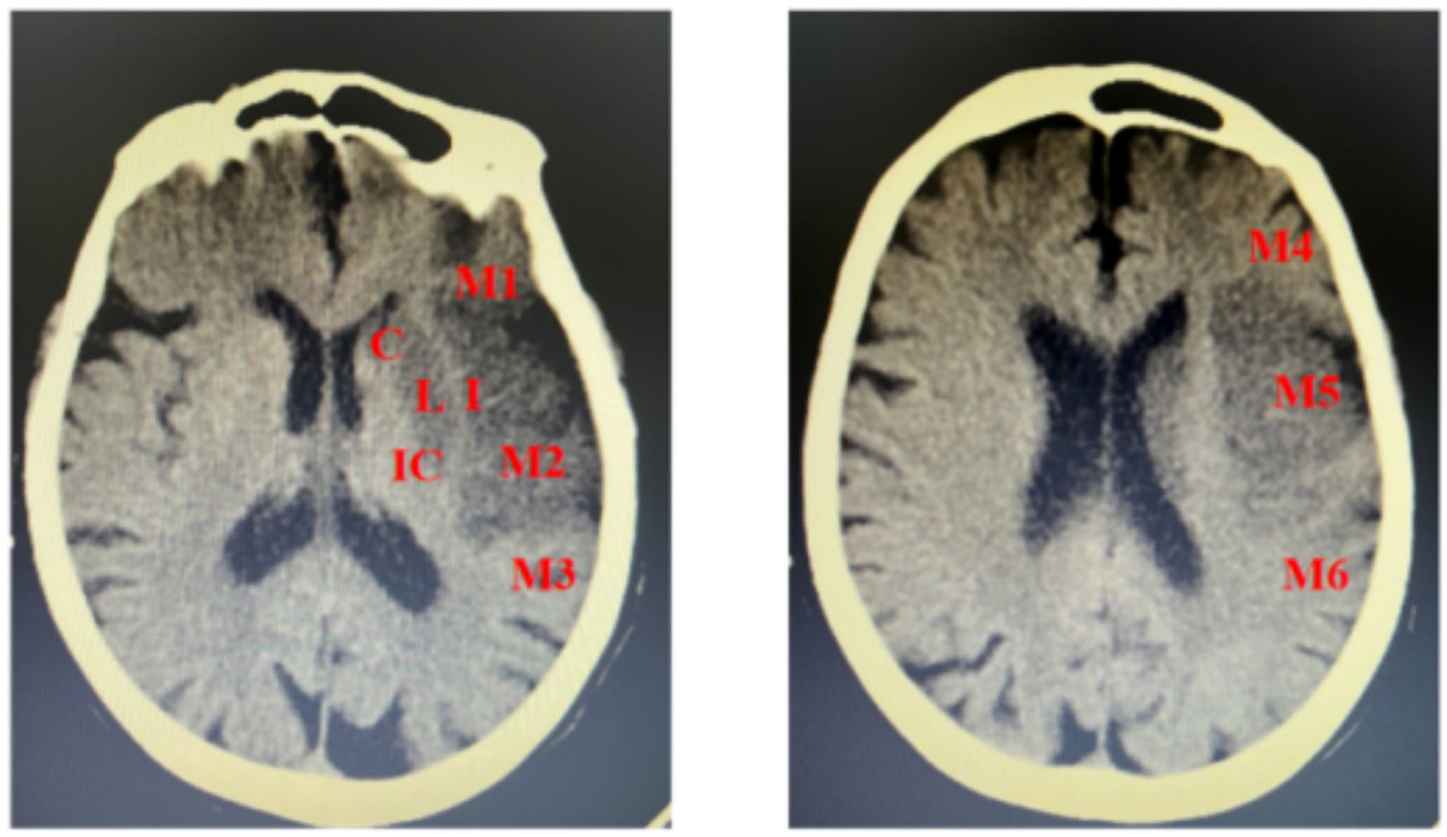
Basal ganglia level (left panel). The level above the basal ganglia (right panel)

All patients underwent ASPECTS scoring based on cranial CT or MRI (MRI was chosen for patients without cranial CT) [13]. The assessment covered ten regions: M1–M6, insular ribbon (I), lentiform nucleus (L), caudate nucleus (C), and the posterior limb of the internal capsule (IC). One point was deducted for each involved region, with the final score being 10^− 1^ × number of involved areas (Fig. 1).

#### Rehabilitation scales

No single measure can describe or predict all aspects of recovery and disability following a stroke. All rehabilitation and outcome scales (e.g., mRS, GCS, NIHSS, VAS, Barthel Index, BBS, Morse Fall Scale) were assessed by three rehabilitation physicians with more than 5 years of clinical experience at two time points: upon hospital admission and at the time of discharge of stroke unit. No post-discharge follow-up was included in this study. The results of these evaluations were recorded in the patients’ medical records. For this retrospective study, the data were extracted from those clinical records. The VAS measures pain intensity; a higher VAS score indicates more severe pain or discomfort (range: 0–10). The GCS is an auxiliary tool for assessing the level of consciousness impairment. The assessment evaluates verbal response, motor response, and eye-opening, a higher GCS score indicates better consciousness (range: 3–15) [14]. The NIHSS is used for the early prediction and systematic evaluation of stroke patients; it is recommended as an effective tool for assessing stroke severity with high reliability, higher NIHSS scores indicate more severe neurological impairment (range: 0–42) [15]. The mRS provides a summary measure of the outcome, with intuitive classification, easy understanding, and covering the entire functional outcome range from asymptomatic to death. It correlates strongly with stroke pathology measures such as infarct volume, higher scores indicate worse functional outcomes (range: 0–6) [16]. The Berg Balance Scale can assess dynamic and static balance and is mainly used for stroke patients. This scale is useful in predicting fall risk and outcomes, as well as assessing the rehabilitation time of hospitalized patients, higher scores suggest better balance ability (range: 0–56) [17]. The BI measures ten basic aspects of self-care and mobility activities and is useful for planning rehabilitation strategies, higher scores reflect better functional independence (range: 0–100) [18]. The Morse Fall Scale is a commonly used assessment tool for predicting the likelihood of patient falls in healthcare facilities, a higher score indicates a higher risk of falling [19]. 

### Statistical analysis

Statistical analysis was conducted using SPSS 22.0 (IBM, Armonk, NY, USA). The data were subjected to normality and homogeneity of variance testing. Continuous data that followed a normal distribution were presented as means ± standard deviations and analyzed using the independent samples t-test. Non-normally distributed continuous data were presented as medians (interquartile ranges) and analyzed using the Mann-Whitney U-test. Categorical data were presented as n (%) and analyzed using the chi-squared test. Multivariable linear regression analyses were performed with rehabilitation scores as the dependent variables and stroke subtype (LAA vs. CE) as the independent variable. Two-sided P-values < 0.05 were considered statistically significant.

## Results

### Characteristics of the patients

A total of 70 patients were included in this study: 44 with the LAA subtype (76.00 (69.75–80.75) years; 50.0% male; 70.45% admitted from emergency department) and 26 with the CE subtype (71.50 (65.25-78.00) years; 57.1% male; 88.46% admitted from emergency department). There were no significant differences between the two subtypes in terms of sex, age, insurance category, smoking, drinking, infarction site, and handedness (all *P* > 0.05) (Table 1).

**Table 1:** Demographic characteristics, lifestyle habits, CT imaging, comorbidities, and complications

Characteristic	Category	CE, *n* = 26	LAA, *n* = 44	χ^2^/Z/T	*P*
Sex, n (%)	Male	13 (50.0)	24 (54.5)	-0.922	0.357
	Female	13 (50.0)	20 (45.5)		
Age, median (IQR), years		76.00 (69.75, 80.75)	71.50 (65.25, 78.00)	-1.989	0.051
Insurance	New Rural Cooperative Medical Scheme	7 (26.9)	9 (20.5)	-0.779	0.436
	Urban Employee Medical Insurance	7 (26.9)	8 (18.2)		
	Urban Resident Medical Insurance	0	5 (11.4)		
	Other Insurance	10 (38.5)	17 (38.6)		
	Self-Pay	2 (7.7)	5 (11.4)		
	> 4.5 h	12 (46.2)	31 (70.5)		
	Unemployed	19 (73.1)	31 (70.5)		
Smoking	Non-smoker	20 (76.9)	29 (65.9)	-1.132	0.258
	Current smoker	2 (7.7)	9 (20.5)		
	Former smoker	1 (3.8)	3 (6.8)		
	Unknown	0	3 (6.8)		
Drinking	Non-drinker	18 (69.2)	35 (79.5)	-0.729 -1.132	0.466 0.258
	Current drinker	6 (23.1)	4 (9.1)		
	Former drinker	2 (7.7)	2 (4.5)		
	Unknown	0	3 (6.8)		
Location of infarction	Left side	9 (34.6)	24 (54.5)	-1.472	0.141
	Right side	16 (61.5)	18 (40.9)		
	Bilateral	1 (3.8)	2 (4.5)		
Handedness	Left	0	0	-0.769	0.442
	Right	26 (100)	44 (100)		
CT volume (cm³)	-	166.50 (113.00, 274.50)	160.50 (107.50, 222.25)	-0.924	0.356
CT-ASPECTS score	-	2.00 (1.00, 4.00)	2.43 (1.00, 5.00)	-0.025	0.980
M1	-	1/0 (16/10)	1/0 (33/11)	-1.179	0.238
M2	-	1/0 (24/2)	1/0 (37/7)	-0.985	0.324
M3	-	1/0 (17/9)	1/0 (33/11)	-0.854	0.393
Caudate nucleus	-	1/0 (13/13)	1/0 (18/26)	-0.735	0.463
Lentiform nucleus	-	1/0 (20/6)	1/0 (29/15)	-0.965	0.335
Insular ribbon	-	1/0 (23/3)	1/0 (34/10)	-1.155	0.248
Posterior limb of the internal capsule	-	1/0 (18/8)	1/0 (26/18)	-0.842	0.400
M4	-	1/0 (16/10)	1/0 (33/11)	-1.179	0.238
M5	-	1/0 (19/7)	1/0 (34/10)	-0.393	0.695
M6	-	1/0 (16/10)	1/0 (30/14)	-0.562	0.574
Stroke unit stay, days	-	20 (5, 31)	18 (6, 30)	-0.273	0.785
Admitted through ER	No	3 (11.54)	13 (29.55)	3.005	0.083
	Yes	23 (88.46)	31 (70.45)		
Hyperhomocysteinemia	Yes	0	8 (18.2)	-2.294	0.022
	No	26 (100)	36 (81.8)		
Diabetes	Yes	4 (15.4)	17 (38.6)	-2.037	0.042
	No	22 (84.6)	27 (61.4)		
Hypertension	Yes	15 (57.7)	28 (63.6)	-0.490	0.624
	No	11 (42.3)	16 (36.4)		
Atrial fibrillation	Yes	22 (84.6)	9 (20.5)	-5.184	< 0.001
	No	4 (15.4)	35 (79.5)		
Pulmonary infection	Yes	18 (69.2)	30 (68.2)	-0.091	0.928
	No	8 (30.8)	14 (31.8)		
Electrolyte disorders	Yes	13 (50.0)	16 (36.4)	-1.111	0.267
	No	13 (50.0)	28 (63.6)		
Urinary incontinence	Yes	10 (38.5)	18 (40.9)	-0.201	0.841
	No	16 (61.5)	26 (59.1)		
Urinary tract infection	Yes	3 (11.5)	5 (11.4)	-0.022	0.982
	No	23 (88.5)	39 (88.6)		
Constipation	Yes	1 (3.8)	1 (2.3)	-0.379	0.705
	No	25 (96.2)	43 (97.7)		

CE: cardioembolism; LAA: large artery atherosclerosis; IQR: interquartile range; CT: computed tomography; ASPECTS: Alberta stroke program early CT score

### CT imaging

There were no statistically significant differences between the two subtypes regarding CT volume (*P* = 0.356) and CT-ASPECTS scores (*P* = 0.980). There were also no significant differences in involvement of the M1–M6 regions, caudate nucleus, lentiform nucleus, insular ribbon, or posterior limb of the internal capsule (all *P* > 0.05) (Table 1).

### Comorbidities and complications

Regarding the comorbidities, the patients with the LAA subtype had higher prevalence of hyperhomocysteinemia (18.2% vs. 0, *P* = 0.022) and diabetes (38.6% vs. 15.4%, *P* = 0.042) than those with the CE subtype, while the patients with the CE subtype had a higher prevalence of atrial fibrillation (84.6% vs. 20.5%, *P* < 0.001), with no significant difference in hypertension (*P* = 0.624) (Table 1). There were no significant differences between the two subtypes regarding complications like pulmonary infection, electrolyte disorders, urinary incontinence, urinary tract infection, or constipation (all *P* > 0.05) (Table 1).

### Symptoms, treatments, outcome, and rehabilitation scale scores

The patients with the two subtypes had no significant differences in muscle strength, sensation, and pathological signs on the hemiplegic side (all *P* > 0.05). There were also no differences in the incidence of hemorrhagic transformation after cerebral infarction and malignant cerebral edema (*P* > 0.05). However, the rate of DHC was higher in patients with the CE subtype compared to those with the LAA subtype (15.4% vs. 2.3%, *P* = 0.041). The patients with the LAA and CE subtypes were comparable regarding thrombectomy, thrombolysis, the outcomes at discharge, and the rehabilitation scores at admission and discharge (Table 2). The multivariable linear regression analysis showed that, after adjusting for confounders, LAA (vs. CE) subtype was independently associated with higher mRS scores (β = 0.86, 95%CI: 0.61–1.22), higher NIHSS (β = 4.85, 95%CI: 0.19–9.89), higher VAS (β = 0.86, 95%CI: 0.66–1.12), and higher GCS (β = 0.62, 95%CI: 0.09-4.00) (all *P* < 0.05) (Table 3).

**Table 2:** Treatment, symptoms, outcome, and rehabilitation scale scores

		CE (*n* = 26)	LAA (*n* = 44)	χ2/Z	*P*
Thrombolysis	Yes	11 (42.3)	3 (6.8)	-1.715	0.086
	No	15 (57.7)	41 (93.2)		
Thrombectomy	Yes	3 (11.5)	3 (6.8)	-0.677	0.499
	No	23 (88.5)	41 (93.2)		
Decompressive hemicraniectomy	Yes	4 (15.4)	1 (2.3)	-2.043	0.041
	No	22 (84.6)	43 (97.7)		
Hemorrhagic transformation after cerebral infarction	Yes	13 (50.0)	17 (38.6)	-0.922	0.357
	No	13 (50.0)	27 (61.4)		
Malignant cerebral edema	Yes	12 (46.2)	13 (29.5)	-1.391	0.164
	No	14 (53.8)	31 (70.5)		
Antiplatelet therapy	Present	7 (26.9)	13 (29.5)	-1.351	0.177
	Absent	19 (73.1)	31 (70.5)		
Rehabilitation consultation	Present	6 (23.1)	17 (38.6)	-1.330	0.184
	Absent	20 (76.9)	27 (61.4)		
Rehabilitation treatment	Present	3 (11.5)	9 (20.5)	-0.950	0.342
	Absent	23 (88.5)	35 (79.5)		
Muscle strength	Non-Grade 0	8 (30.8)	22 (50.0)	-1.430	0.153
	0 Grade	16 (61.5)	19 (43.2)		
	Upper/Lower Limb 0/Non-Grade 0	2 (7.7)	3 (6.8)		
Sensation	Normal	5 (19.2)	11 (25.0)	0.739	0.460
	Other	1 (3.8)	2 (4.5)		
	Uncooperative	20 (76.9)	31 (70.5)		
Pathological signs	Positive	15 (57.7)	23 (52.3)	-0.437	0.662
	Negative	10 (38.5)	19 (43.2)		
	Other	1 (3.8)	2 (4.5)		
Outcome	Improved	18 (69.2)	29 (65.9)	-0.289	0.773
	Discharged	7 (26.9)	13 (29.5)		
	Death	1 (3.8)	2 (4.5)		
mRS	-	0.00 (0.00, 0.00)	0.00 (0.00, 0.00)	-0.820	0.412
Berg	-	0.00 (-0.25, 2.00)	0.00 (0.00, 3.00)	-0.603	0.547
BI	-	0.00 (-7.50, 13.75)	0.00 (-15.00, 5.00)	-1.176	0.240
Morse	-	0.00 (-10.00, 16.25)	0.00 (-15.00, 15.00)	-0.191	0.848
NHISS	-	0.00 (-3.25, 2.50)	0.00 (-3.75, 2.75)	-0.000	> 0.999
GCS	-	0.00 (-2.25, 3.00)	0.00 (-3.50, 3.00)	-0.049	0.961

CE: cardioembolism; LAA: large artery atherosclerosis; mRS: modified Rankin scale; BBS: Berg Balance Scale; BI: Barthel index; Morse: Morse Fall Risk Scale; NIHSS: National Institutes of Health Stroke Scale; GCS: Glasgow Coma Scale

**Table 3:** Multivariable linear regression analysis for rehabilitation scale scores

Rehabilitation scale scores	LAA vs. CE (ref.)	
	Crude adjustment (β, 95% CI)	Adjusted (β, 95% CI)
mRS score	0.92 (0.63, 1.33)	0.86 (0.61, 1.22) ^a^
Berg score	0.05 (0.00, 12.25)	0.04 (0.00, 7.60) ^b^
BI score	0.00 (0.00, 10.26)	0.510 (0.000, 9.897) ^b^
Morse score	0.40 (0.00, 10162.10)	1.74 (0.00, 35191.79) ^d^
NHISS	0.85 (0.66, 1.10)	4.85 (0.19, 9.89) ^e^
VAS	0.85 (0.66, 1.10)	0.86 (0.66, 1.12) ^f^
GCS	0.78 (0.09, 6.96)	0.62 (0.09, 4.00) ^g^

CE: cardioembolism; LAA: large artery atherosclerosis; CI: confidence interval; mRS: modified Rankin scale; BBS: Berg Balance Scale; BI: Barthel index; MFS: Morse Fall Risk Scale; NIHSS: National Institutes of Health Stroke Scale; VAS: visual analogue scale; GCS: Glasgow Coma Scale

^a^: Adjusted for cost category, cerebral infarction site, M3, stroke unit, diabetes, malignant cerebral edema, antithrombotic therapy, muscle strength, sensation, pathological signs

^b^: Adjusted for sex, cost category, smoking, alcohol consumption, caudate nucleus C, pulmonary infection, rehabilitation, sensory, pathological signs

^c^: Adjusted for smoking, drinking, lentiform nucleus L, stroke unit, atrial fibrillation, bone removal, malignant cerebral edema, rehabilitation, muscle strength, pathological signs

^d^: Adjusted for smoking, lentiform L, M6, stroke unit, hypertension, urinary tract infection, rehabilitation treatment

^e^: Adjusted for duration from onset to visit, cost category, smoking, alcohol consumption, CT2, ASPETS, M1, M3, caudate nucleus C, lentiform nucleus L, stroke unit, bone removal, malignant cerebral edema, rehabilitation, muscle strength

^f^: Adjusted for time from onset to visit, cost category, smoking, cerebral infarction site, ASPETS, insula I, internal capsule hindlimb IC, M5

^g^: Adjusted for sex, time from onset to visit, cost category, smoking, alcohol consumption, cerebral infarction site, caudate nucleus C, lentiform nucleus L, stroke unit, diabetes, urinary incontinence, malignant cerebral edema, antithrombotic therapy, rehabilitation therapy, muscle strength

## Discussion

The findings of this study showed that hyperhomocysteinemia and diabetes were more common in patients with the LAA subtype of LHI in the MCA, while atrial fibrillation and decompressive craniectomy were more common in patients with the CE subtype of LHI in MCA. CE and LAA subtypes may differentially impact rehabilitation outcomes in patients with LHI. The study highlights distinct risk factors and treatment implications for the LAA and CE subtypes of LHI in the MCA, underlining the importance of subtype-specific management strategies for improved patient outcomes.

The results showed that patients with CE LHI were more likely to undergo DHC, a procedure generally used to reduce mortality caused by malignant cerebral edema [20]. Observational data suggest that patients with CE LHI may develop malignant cerebral edema earlier than those with LAA LHI, although the underlying mechanisms remain unclear. Nevertheless, Xie et al. [21] reported a higher rate of CE than LAA in patients with malignant cerebral edema. A previous study showed that the more acute the onset of stroke, the more difficult it becomes to establish collateral circulation, resulting in relatively more severe damage to brain function [22]. In addition, CE LHIs are often sudden, while the formation of the infarction area in LAA LHI is relatively slow [23]. Therefore, combining literature and study results, we speculate that the incidence of malignant cerebral edema in patients with LAA LHI is lower than in those with CE LHI, which may be related to the relatively slower onset of LAA strokes and the easier formation of collateral circulation. The multivariable linear regression analysis also suggests that the mRS, NIHSS, VAS, and GCS scores were significantly different between LAA and CE, independent of confounding variables. The analysis demonstrated that, compared to the CE subtype, the LAA subtype was independently associated with higher mRS, NIHSS, and VAS scores, as well as higher GCS scores, indicating worse functional outcomes, greater stroke severity and pain, but better consciousness level. Among the findings, only the positive association between LAA and higher GCS scores, which indicated better consciousness level, supporting the known worse prognosis of CE compared with LAA [24]. However, the findings on mRS, NIHSS, and VAS scores warrant further investigation.

This study found that patients with LAA LHI were more likely to have diabetes and hyperhomocysteinemia than those with CE LHI. Diabetes can promote the opening of collateral circulation in LHI caused by acute occlusion of the internal carotid artery [25]. In line with this, our study found that LAA LHI was more likely to be associated with diabetes. Previous studies suggest that chronic diabetes may promote collateral vessel development through mechanisms such as VEGF upregulation, potentially aiding perfusion in large artery atherosclerotic infarctions [26, 27]. In our cohort, LAA-related LHI was more commonly associated with diabetes, possibly reflecting this compensatory vascular remodeling. Although hyperglycemia can worsen edema in patients with cerebral ischemia [28–30], no significant differences were observed in functional scores between the two subtypes. Some patients classified as LAA had documented atrial fibrillation (AF); however, AF was determined to be non-causative based on an integrated assessment following the TOAST classification criteria. The TOAST system emphasizes identifying the most probable cause of stroke through clinical presentation, cardiac evaluation, and neuroimaging. An insidious symptom onset, evidence of atherosclerotic plaque at typical locations (e.g., origin of the internal carotid artery or M1 segment of the MCA), flame-shaped occlusion, presence of abundant collaterals, and lack of left atrial/appendage thrombus on echocardiography all supported LAA as the primary etiology in these cases, rather than cardioembolism. This approach is consistent with current recommendations and literature on determining stroke mechanisms in patients with coexisting AF and atherosclerosis [7, 31–33]. 

Hyperhomocysteinemia is associated with atherosclerosis and stroke, although it is not clear whether there is a causal relationship. Elevated homocysteine levels are associated with an increased likelihood of acute cerebral infarction, and elevated serum homocysteine is considered an independent risk factor for stroke in patients with diabetes [8, 34–36]. Therefore, in high-risk stroke populations, active intervention for patients with hyperhomocysteinemia and/or diabetes may have some significance in preventing the development of LAA LHI. On the other hand, atrial fibrillation was more common in patients with CE LHI, in accordance with the known risk factors for CE stroke [37]. Hypertension is associated with a higher risk of stroke [38], although no significant differences were observed between the two subtypes in this study. A previous study showed that hypertension was protective against malignant cerebral edema [21], while another showed the contrary [39]. Additional studies are necessary.

The study has several limitations. First, patients were recruited from a single hospital over a limited time period, yielding a relatively small sample size. Furthermore, this small sample may have contributed to the unexpected finding that the LAA subtype was independently associated with higher mRS, NIHSS, and VAS scores—indicating worse functional outcomes, greater stroke severity, and increased pain. Furthermore, the different thresholds for the LHI may also lead to bias. In addition, because they were from a single center, there is a risk of bias due to local practices and policies. This study utilized a retrospective cohort design, the limitations inherent to retrospective analyses apply, including potential bias and limited control over confounding variables. Prospective multicenter studies are warranted.

## Conclusion

In conclusion, this study revealed differences in the rates of DHC between patients with CE LHI vs. those with LAA LHI, suggesting new considerations for clinical decision-making. Furthermore, there were differences in diabetes, atrial fibrillation, and hyperhomocysteinemia between the two subtypes. CE and LAA subtypes may differentially impact rehabilitation outcomes in patients with LHI. These differences may be related to malignant cerebral edema in LAA and CE LHI, offering potential insights into the management of LHI. Nevertheless, these findings require further confirmation through prospective, multicenter studies with larger sample sizes.

## Electronic supplementary material

Below is the link to the electronic supplementary material.

Supplementary Material 1

## Data Availability

All data generated or analysed during this study are included in this published article and its Supplementary Information files.

## Abbreviations

CE: Cardioembolic
LHI: Large hemispheric infarction
MCA: Middle cerebral artery
LAA: Large artery atherosclerosis
DHC: Decompressive hemicraniectomy
SAO: Small artery occlusion
TOAST: Trial of ORG 10172 in acute stroke treatment
mRS: modified rankin scale
BBS: Berg balance scale
BI: Barthel index
MFS: Morse fall risk scale
GCS: Glasgow coma scale
NIHSS: National institutes of health stroke scale
VAS: Visual analog scale
CT: Computed tomorgraphy
SAO: Small artery occlusion
ASPECTS: Alberta stroke program early CT score
IQR: Interquartile range
