# Supplementary figures and images for: Large artery atherosclerotic versus cardioembolism subtypes of large hemispheric infarction in the middle cerebral artery
Source: Neurol Sci. 2025 Jul 12;46(10):5173–81. doi: 10.1007/s10072-025-08347-9 (PMC12488826; doi:10.1007/s10072-025-08347-9)

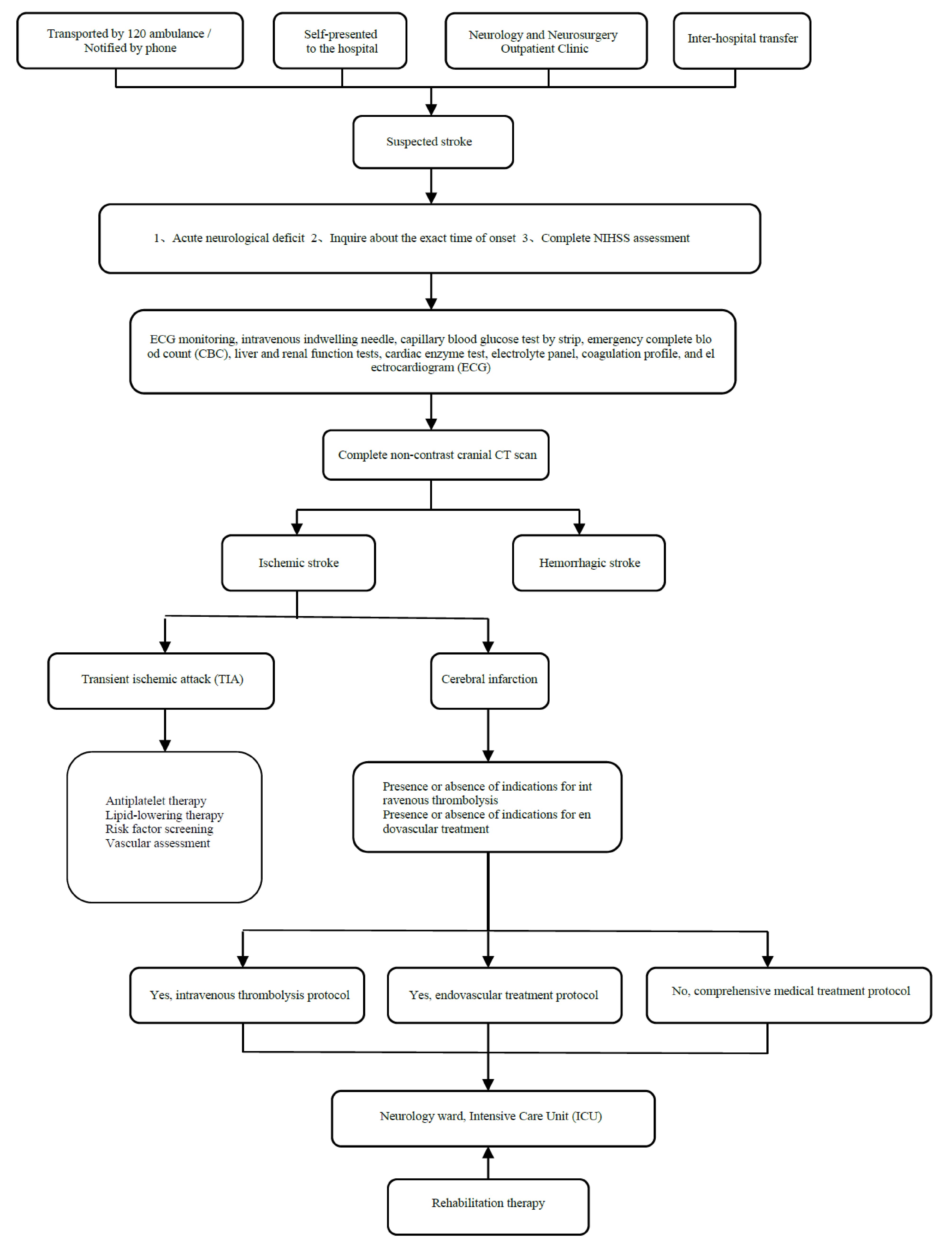

Supplement: Supplementary file 1 — Supplementary Material 1 [file 10072_2025_8347_MOESM1_ESM.jpg]
